# Biologically Inspired Scene Context for Object Detection Using a Single Instance

**DOI:** 10.1371/journal.pone.0098447

**Published:** 2014-05-28

**Authors:** Changxin Gao, Nong Sang, Rui Huang

**Affiliations:** Key Laboratory of Ministry of Education for Image Processing and Intelligent Control, School of Automation, Huazhong University of Science and Technology, Wuhan, China; Huazhong University of Science and Technology, China

## Abstract

This paper presents a novel object detection method using a single instance from the object category. Our method uses biologically inspired global scene context criteria to check whether every individual location of the image can be naturally replaced by the query instance, which indicates whether there is a similar object at this location. Different from the traditional detection methods that only look at individual locations for the desired objects, our method evaluates the consistency of the entire scene. It is therefore robust to large intra-class variations, occlusions, a minor variety of poses, low-revolution conditions, background clutter etc., and there is no off-line training. The experimental results on four datasets and two video sequences clearly show the superior robustness of the proposed method, suggesting that global scene context is important for visual detection/localization.

## Introduction

Visual detection/localization is one of the basic functions of human vision. Most object detection methods are learning-based, which use lots of positive and negative samples to train a classifier. Training is often a time-consuming process and the training samples are very difficult to collect and manually label in many domains. Training-free image analysis methods are becoming more and more popular in recent years [1], [2]. This paper addresses the problem of object detection in the training-free setting, namely, object category detection using only a single instance.

As a classical training-free computational model, template matching is very effective for object localization, by computing point-to-point correlation of a model pattern with the image pattern. It has been widely used in object detection, object tracking, feature detection, and motion estimation, etc. However, this technique is designed for object *instance* detection, i.e., searching only for the same instance as the template, because single templates are inadequate for encoding large intra-class variations, and local appearance features have limited robustness to occlusion, pose, low-resolution conditions and background clutter. The human visual system is remarkably robust and can work effectively even under highly uncertain and ambiguous conditions, where contextual information plays a critical role [3]–[5]. This paper focuses on improving the single instance based object detection method using contextual information.

Given the excellent performance of human vision in this task, it is reasonable to look to biology for inspiration. After surveying the relevant psychophysical and neurophysiological research, we find that *scene-centered viewpoint* [6]–[8] and *change blindness* phenomenon [9], [10] may provide help to this task. Scene-centered viewpoint suggests that humans can capture the abstract meaning of a real world scene only using their global spatial layout without necessarily knowing the objects in the scene. Change blindness suggests that human often fail to notice the relatively large changes to visual scenes.

### Scene-centered viewpoint

Traditional conceptions of research in computer vision treat objects as the atoms of recognition. In contrast, some experimental studies have suggested that the recognition of real world scenes may be initiated from the encoding of the global configuration, without necessarily identifying the objects they contain [4], [7], [11], [12]. Humans are able to comprehend the amount of perceptual and semantic information (the semantic category of each image as well as a few objects and their attributes) in less than 200 milliseconds, which is referred to as getting the gist of the scene [8], [13], [14]. Biederman has demonstrated human identify a scene as quickly and as accurately as a single object [15]. The semantic category of most real world scenes can be inferred from their spatial layouts [6], [12], [16], [17], [18]. These experiments in scene perception and visual search suggest that the visual system first processes contextual information in order to index object properties. Following these results, some computer vision methods recognize an image using global context representation, termed as context-based approaches.

### Change blindness

Humans often fail to notice the large changes to visual scenes, called change blindness. Several studies on change blindness have demonstrated that subjects can be totally blind to object changes (such as displacement and suppression) even when these changes affect meaningful parts of the scene [18]: observers frequently fail to notice when the central actor in a simple motion picture is replaced by a different person wearing different clothing and when the person they are talking to is surreptitiously replaced by a different person [10], [19]; observers successfully recognize a previously attended object on a memory test even when they have failed to detect a change to that object [20]; observers can remain the pre- and post-change information at better than chance levels, even when they have failed to detect the change [21]. These studies suggest that human representations of visual scenes are sparse and incomplete [9], [22]. It should be noted that the “large changes” in these studies indicate that objects are replaced with another object instance from the same category.

**Figure 1:**
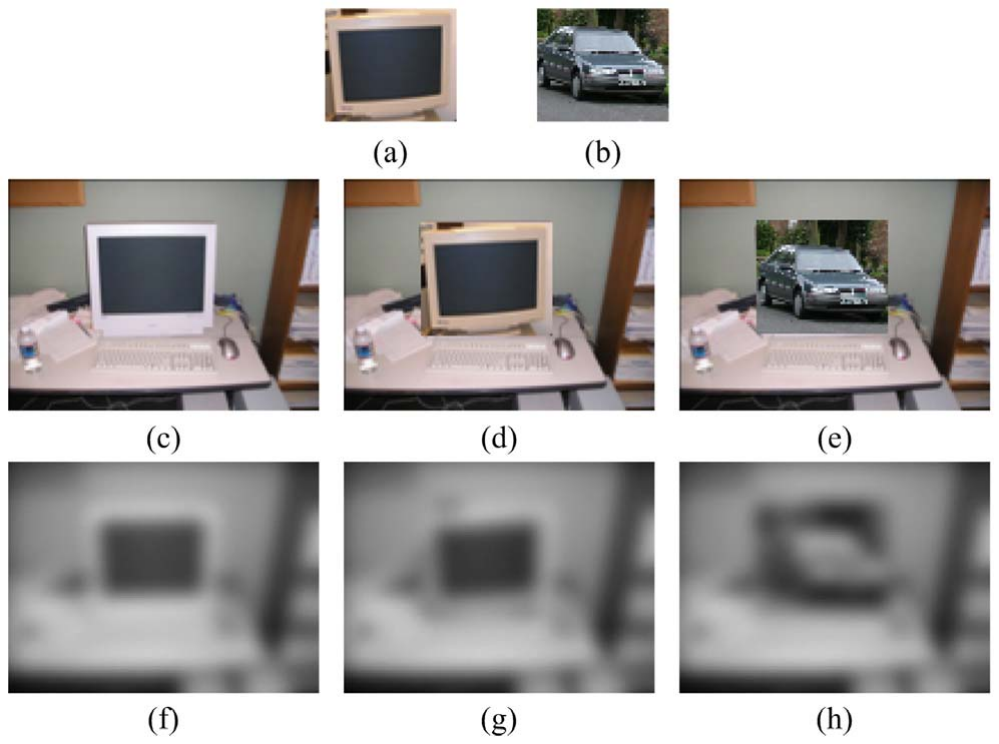
Illustration of the variety of coarse scene layout (produced using Gaussian blur with 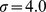) by replacing object instances in the scene with templates (another object instance). All the scene images are from the LabelMe database [25]. (a) and (b) are monitor and car templates respectively; (c) shows a scene which contains a monitor; (f ) shows the layout of scene shown in (c); (d) and (e) show the results after replacing the monitor in the scene with the two templates; (g) and (h) show the scene layouts corresponding to images with replaced instances.

The motivation inferred from these two biological findings is that the spatial configuration of a scene is very important to human visual perception, and sufficiently big changes will break the structure of the scene while smaller changes will not. The change in global spatial layout is imperceptible when an object instance in a scene is replaced by another instance (at the same scale) from the same category, while it is more pronounced when replacement happens between distinct categories, as shown in Fig. 1. We use the blurred images as the coarse layout of the scenes. Despite the monitor template contains a small amount of background contents and the resulting images Fig. 1(c) and Fig. 1(d) are noticeably different, the layouts are very similar. The correlation coefficient (gray values are used) between Fig. 1(f) and Fig. 1(g) is 0.98 and the one between Fig. 1(f) and Fig. 1(h) is 0.76. It illustrates that the change in global spatial layout is imperceptible when the replacement happens between the instances within the same category, and it is sensitive to those replacements between distinct categories.

**Figure 2:**
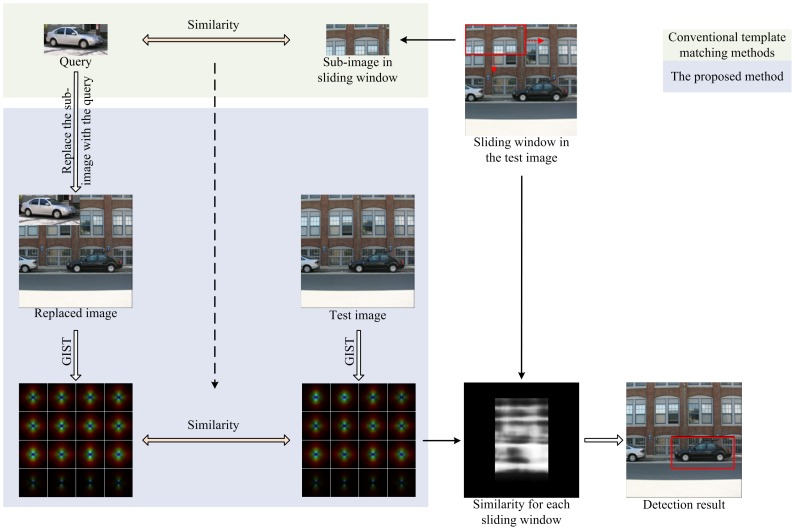
Sketch of the difference between conventional template matching and our template matching. Conventional template matching methods measure the similarity of the template and the sub-window; while our template matching method measures the similarity of the scene image; and the new scene image with the replaced window. All the scene images are from the LabelMe database [25], and the template is cropped from an image also in the LabelMe dataset.

To this end, we present a single instance based object category detection method using global scene context, which uses the constraints of scene context to improve the performance of matching (Preliminary version of portions of this work has been published in [23]). This method is quite different from conventional template matching methods: conventional methods measure the similarity of a query template and a sub-image using local features within them, while our method measures the compatibility of the two by replacing the sub-image with the template and evaluating the change in the scene context. The differences between the two methods are illustrated in Fig. 2.

To achieve this goal we have to consider how to measure the global scene context appropriately. The representation of the global scene is expected to capture the main layout of the scene. Holistic representations of scenes, termed as the scene-based representations, have been proved to be an efficient method for extracting coarse layout information [24]. Global representation methods are more robust than local appearance representations, because they bypasses some challenging problems, such as local feature detection, feature grouping, and object recognition, which are necessary processing steps in object-based representations. Performance of these intermediate steps has a direct impact on the robustness of the local feature based matching methods. Consequently, we adopt a global scene-based representation method to describe a scene image.

There are three key contributions in this work. First, we argue that traditional template matching is instance-level detection and cannot account for large intra-class divergence, and therefore we propose to use global scene context to improve simple template matching. Second, inspired by biological findings, in particular, scene-centered viewpoint and change blindness phenomenon, we propose to measure global scene context using consistency of spatial layout, which is described by the scene-based representations in this work. Third, we propose a scheme to detect a category of objects using a single instance as query by replacing the sliding windows with the query and measuring the change in the global context. The smaller the change in global context is, the more likely there is a similar object at the replaced location. To evaluate the robustness of our method to large intra-class variations, occlusions, a minor variety of poses, low-resolution conditions, background clutter, we perform a detection/localization task on the Pascal VOC 2008 dataset [25], USC sequence dataset [26], LabelMe dataset [27], UIUC car dataset [28], and two videos. The promising results demonstrate the effectiveness of the proposed method.

#### Related Works

This paper presents a biologically-inspired training-free object detection method using scene context. Some related research are introduced in this section. First, we present conventional template matching methods. Second, we introduce scene-based representations. Finally, we present some approaches that use context to improve computer vision methods

#### Template matching methods

Conventional template matching has two disadvantages: sensitivity to rotation and computational cost. Many improvements to template matching therefore mainly focus on these two aspects: Ullah and Kaneko [29] have presented a rotation-invariant template matching approach using gradient information in the form of orientation codes as the feature; Yoshimura and Kanade [30] have presented a fast computation method of the normalized correlation for multiple rotated templates by using multi-resolution eigenimages; Choi and Kim [31] have proposed a rotation invariant template matching algorithm based on the combination of the projection method and Zernik moments; Vanderbrug and Rosenfeld [32] have reduced computationally cost by initially using only a sub-template, and applying the rest of the template only when the sub-template's degree of match exceeds a threshold; Krattenthaler et al. [33] have proposed a reduced-cost correlation template matching approach where matching is not performed with the entire template but with a pre-computed set of points of the template.

All the enhancements of template matching are very effective. However, to go beyond instance level detection, i.e., to use a single template to find a broader category of objects, these methods are still too sensitive to large intra-class variations, occlusion, poses and low-resolution conditions, because of the limited robustness of the local appearance features that form the template.

#### Scene-based representations

Scene-based representations consider scene-images as a whole and describe them as low-dimensional features. Such representations are robust, because (1) they bypass some challenging problems, such as segmentation, feature grouping, and object recognition, which are necessary processing steps in object-based representations; and (2) they average out the local noise by processing globally [24]. The challenge to discover a compact and holistic representation for unconstrained images has prompted significant recent research. The semantic category of most real world scenes can be inferred from their spatial layouts (e.g. a spatial arrangement of the objects and the scene ground plane [16], [17]; an arrangement of basic geometrical forms such as simple Geon clusters [6]; or the spatial relationships between regions or blobs of particular size and aspect ratio [11], [12], [17]). Torralba et al. [34] encode some spatial information using steerable wavelet pyramids within individual image sub-regions on a regularly-spaced grid; whilst Siagian and Itti [24] use a multi-scale set of early-visual features instead of steerable wavelet pyramids. Oliva and Torralba [18] introduced a representation for describing the structure of real world scenes, called Spatial Envelope, which is described as a set of perceptual properties (naturalness, openness, roughness, ruggedness and expansion).

In our scene context based detection method, how to represent the scene context appropriately is very important. Scene-centered representations, which are more robust than local appearance representations, may help in this regard. “*Gist*” [34], which extracts coarse layout information of a scene, is used to represent scene images in this work.

#### Context-based methods

The relationship between environments and the objects within these environments is very strong in real-world scenes. In typical visual-search experiments, the context of a target is a great hindrance to the detection process [35]. However, the context provides rich information that can serve to help the recognition and detection process [35]. Context-based approaches to object recognition have become increasingly popular over the years. There are three key context representations at different levels: neighbor-based context, object-based context and scene context. Note that, the level of context we used in this paper is scene context. And Colleguilas and Belangie [36] review a variety of approaches for context-based object categorization.

Many context-based methods have demonstrated that context information can be used to improve aspects of computer vision, such as image segmentation, visual attention [37], [38], object detection [39], object recognition [40], [41], visual tracking [42], [43], and scene understanding [38], [44], [45]. Thus, it is reasonable to apply context to improve template matching methods.

## Methods

**Figure 3:**
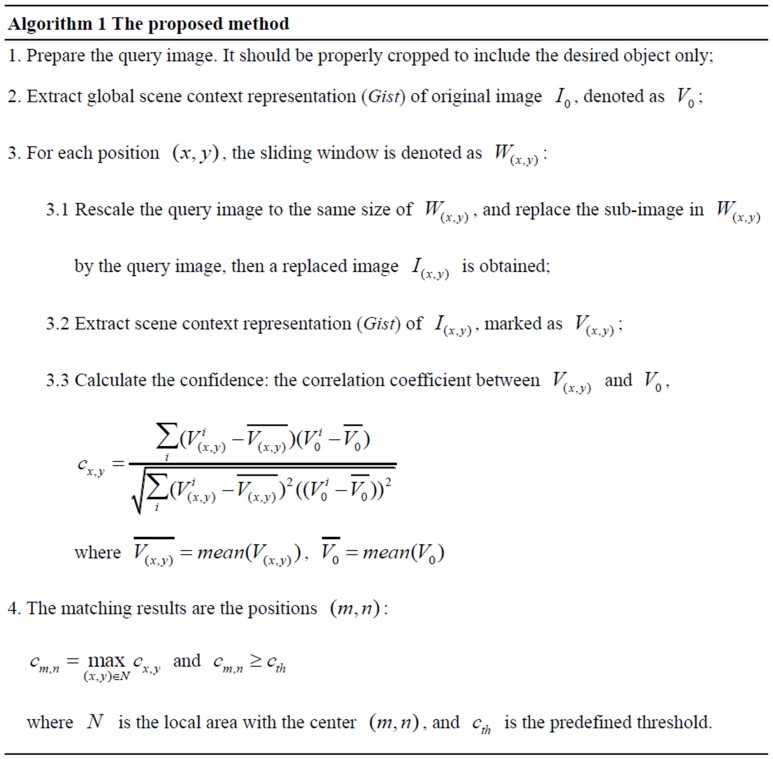
The flowchart of the proposed algorithm.

To locate all instances of an object of interest in a scene image, we slide windows across all positions in the image, as do most object detection approaches. Instead of measuring the similarity of the query and the sub-image in each window directly, we replace the sub-image with the query and measure the change in the global context, as shown in Fig. 2. Then we measure the confidence in how much the query image matches a sub-image using the similarity between the global scene context of the original scene and the replaced scene. Fig. 3 shows the major stages of the proposed method. The main difference between the proposed method and conventional template matching is that traditionally only local information is used for matching, while the proposed method uses global scene context for matching. Due to the ability of global scene context to describe the coarse layout information, the proposed method, to a certain extent, can be considered as a global layout guided matching method.

In this work, *Gist* [18] is applied as the global scene representation. An input image 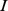 is filtered using a set of Gabor filters with 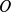 orientations and 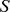 scales. For each Gabor filter 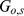, (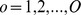, 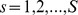), the filtered map can be computed by 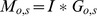. *Gist* cumulates information in an 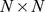 grid of sub-regions over the maps. Each element of the *Gist* vector is computed by taking the sum over a corresponding sub-region (specified by indices 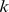 and 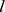 in the horizontal and vertical direction respectively, 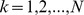, 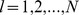):
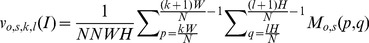
(1)where 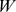 and 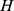 are the width and height of the entire image, 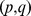 is position index. The final 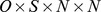 *Gist* vector is composed of all the elements: 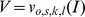.

## Results and Discussion

### Experimental Design

We experimentally compare our method against a conventional template matching method, by performing training-free car detection tasks on four well-known datasets: Pascal VOC sets of 2008 [25], USC sequence (one sequence of volume four in USC-SIPI image dataset [26]), LabelMe [27], UIUC [28]. In addition, we test our method on two videos to evaluate a detection-based tracking task. Objects in these datasets can be highly occluded, occupy various poses, include various types within the same category, be low-resolution conditions or have cluttered background. The robustness of detection performance and the generalization of our method are evaluated for items of each dataset (listed in Table 1). Finally, we evaluate the effect of different templates for our method. To sum up, we perform 6 groups of experiments: Experiments on PASCAL, Experiments on USC sequence, Experiments on LabelMe, Experiments on UIUC, Experiments on two videos sequences, and Evaluation of generalization ability (the effect of the different query templates).

**Table 1:** Evaluated items of the four datasets.

Dataset	Evaluating items
Pascal VOC 2008	robustness to occlusions, variety of poses, intra-class variations, and background clutter
USC sequence	robustness of occlusions and variety of poses
LabelMe & UIUC	detection performance for both 100% and 25% resolution, generalization
Two videos	non-rigid transform, occlusions, low-resolution

Three methods are compared in our experiment on these datasets: (1) template matching method, correlation coefficient of gray values of query and sub-image in a sliding window is used to measure their similarity; (2) local *Gist* based template matching, correlation coefficient of *Gist* of query and sub-image in a sliding window is used to measure their similarity; (3) the proposed method.

*Gist* descriptor is applied as the global scene representation. In our experiments, a bank of Gabor filters are constructed using 8 orientations and 4 scales, in a total of 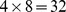 filters. The image is divided into a 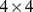 grid, and the average energy of each channel in each gird cell is computed. Then the resulting image representation is a 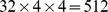 dimensional feature vector.

In our experiments, the sliding sub-window moves in steps of 5 pixels horizontally and 2 vertically on all these databases. After thresholding the confidences of all positions, “repeated part elimination” [28] algorithm is used to obtain the final positions.

In addition, we performed all processing in grayscale, even when color images are available. Our approach can be easily expanded to a multiple-scale detection task by searching at each scale. However, to highlight the distinguished performance of our method, we just focus on the single scale situation in this work. Thus, the testing images were rescaled before localization/detection so that the car instances are roughly the same size.

### Experiments on PASCAL

**Figure 4:**
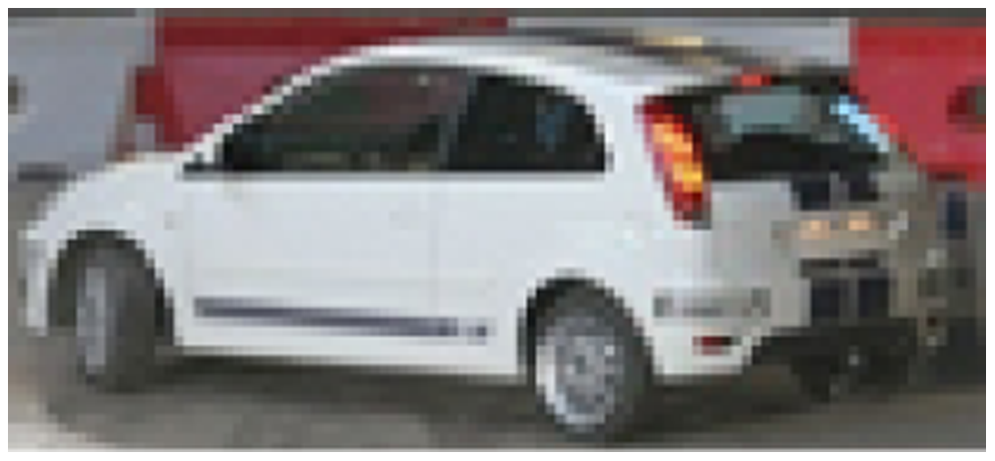
The query image used in the experiments on the Pascal VOC 2008. The size of the query image is 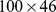 pixels. The query is cropped from a different image in the Pascal VOC database.

**Figure 5:**
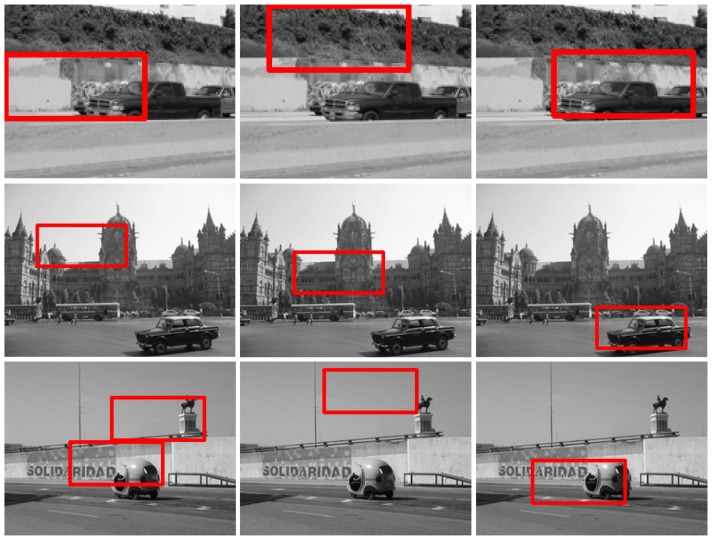
The car detection results with large intra-class variance on the Pascal VOC 2008. For each row, the left image is the result of template matching; the middle image is the results of local *Gist* based template matching; and the right image is the results of the proposed method.

**Figure 6:**
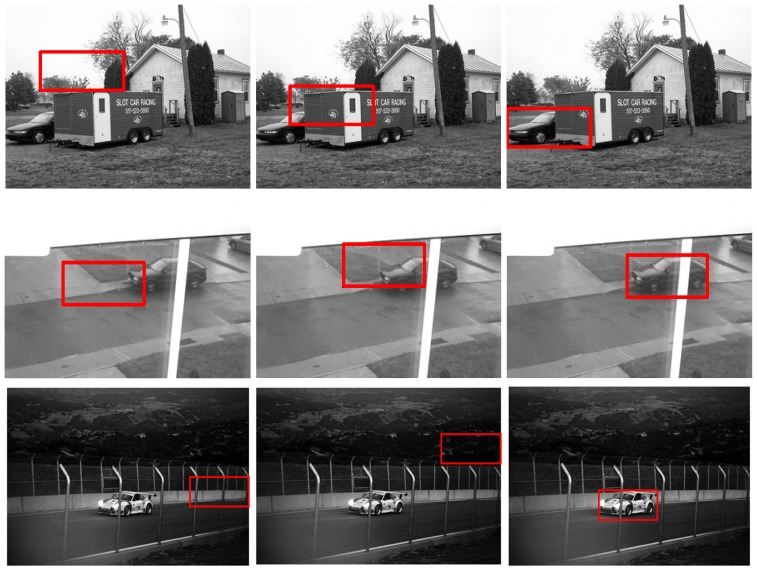
The car detection results with occlusion on the Pascal VOC 2008. For each row, the left image is the result of template matching; the middle image is the results of local *Gist* based template matching; and the right image is the results of the proposed method.

**Figure 7:**
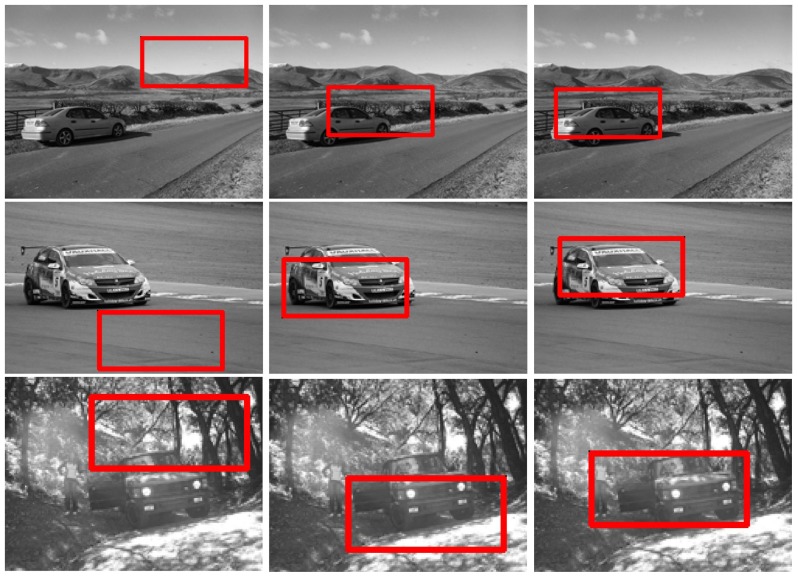
The car detection results with variety of poses on the Pascal VOC 2008. For each row, the left image is the result of template matching; the middle image is the results of local *Gist* based template matching; and the right image is the results of the proposed method.

**Figure 8:**
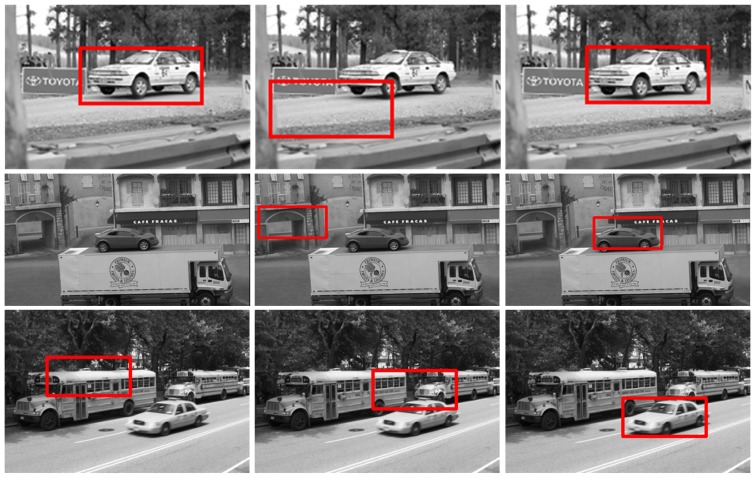
The car detection results with background clutter on the Pascal VOC 2008. For each row, the left image is the result of template matching; the middle image is the results of local *Gist* based template matching; and the right image is the results of the proposed method.

In this subsection, we evaluate the robustness of our method to large intra-class variations, occlusions, poses and background clutter on the Pascal VOC 2008 dataset. Note that the challenges in each test image are often intertwined. For example, in a testing image, the car may be quite different from the template in both appearance and pose. We report 3 testing results for each challenge. Fig. 4 presents the template used in the experiments. Fig. 5, Fig. 6, Fig. 7, and Fig. 8 show the matching results with large intra-class variations, occlusions, poses, and background clutter respectively. The results of these challenges demonstrate the robustness and effectiveness of the proposed method.

### Experiments on USC sequence

**Figure 9:**
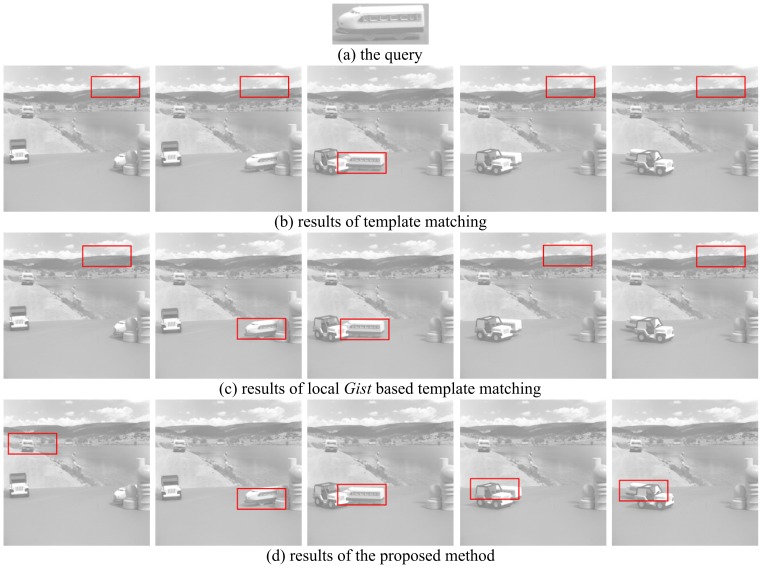
The object detection results of Group 1 on the USC sequence, using the three methods: template matching, local *Gist* based template matching and the proposed method. Five frames of test results are presented, which are Frame 2, 3, 6, 7, 8 of original sequence. (a) shows the query image in the experiments, which is cropped from frame 5; (b) shows the results of template matching; (c) shows the results of local *gist* based template matching; (d) shows the results of the proposed method. For (b), (c) and (d), each image is the detection result for a specific frame.

**Figure 10:**
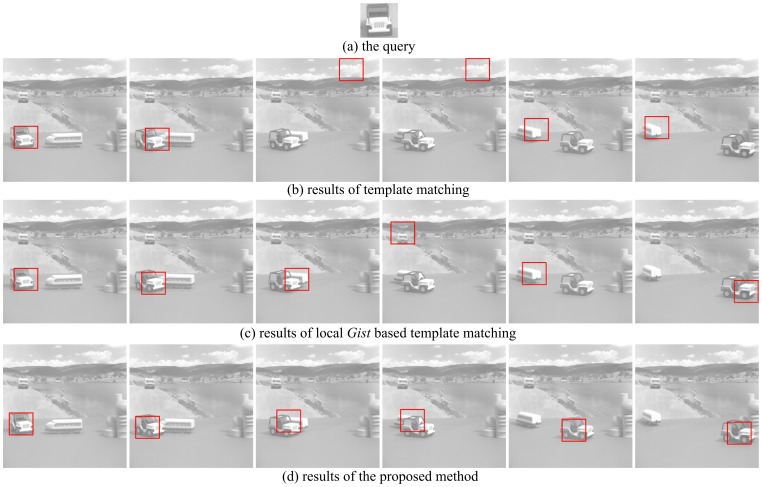
The object detection results of Group 2 using the three methods: template matching, local *Gist* based template matching and the proposed method. Six frames testing results are presented, which are Frame 5, 6, 7, 8, 9, 10 of original sequence. (a) shows the query image in the experiments, which is cropped from frame 5; (b) shows the results of template matching; (c) shows the results of local *Gist* based template matching; (d) shows the results of the proposed method. For (b), (c) and (d), each image is the detection result corresponding to a frame.

For in-depth analysis of our method, we evaluate the robustness to occlusion and variety of poses on a USC sequence, which contains three cars, consists of 10 frames with 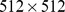 pixels. The challenge of the first group of experiments is occlusion. Five frames with occlusion are used for testing. The second group of experiments investigates the effect of poses. Six frames with a wide variety of poses are tested. The results of these two groups of experiments are shown in Fig. 9 and Fig. 10 respectively. The template matching method, local *Gist* based template matching and our method are compared. The numbers of correctly detected frames are presented in Table 2 for both groups. It can be seen that only one error occurs for our method. The experimental results on the USC sequence show that our proposed method is more robust to occlusion and variety of poses.

**Table 2:** The Number of correct detected frames with the three methods for the two groups of experiments on the USC sequence.

Methods	Group 1	Group 2	Missed frames
Template matching	1	2	8
Local *Gist* based template matching	2	4	5
The proposed method	4	6	1

For Group 1 experiments, the results of 5 frames are presented and 6 frames for Group 2. The numbers of missed frames of the three methods are also given.

### Experiments on LabelMe

**Figure 11:**
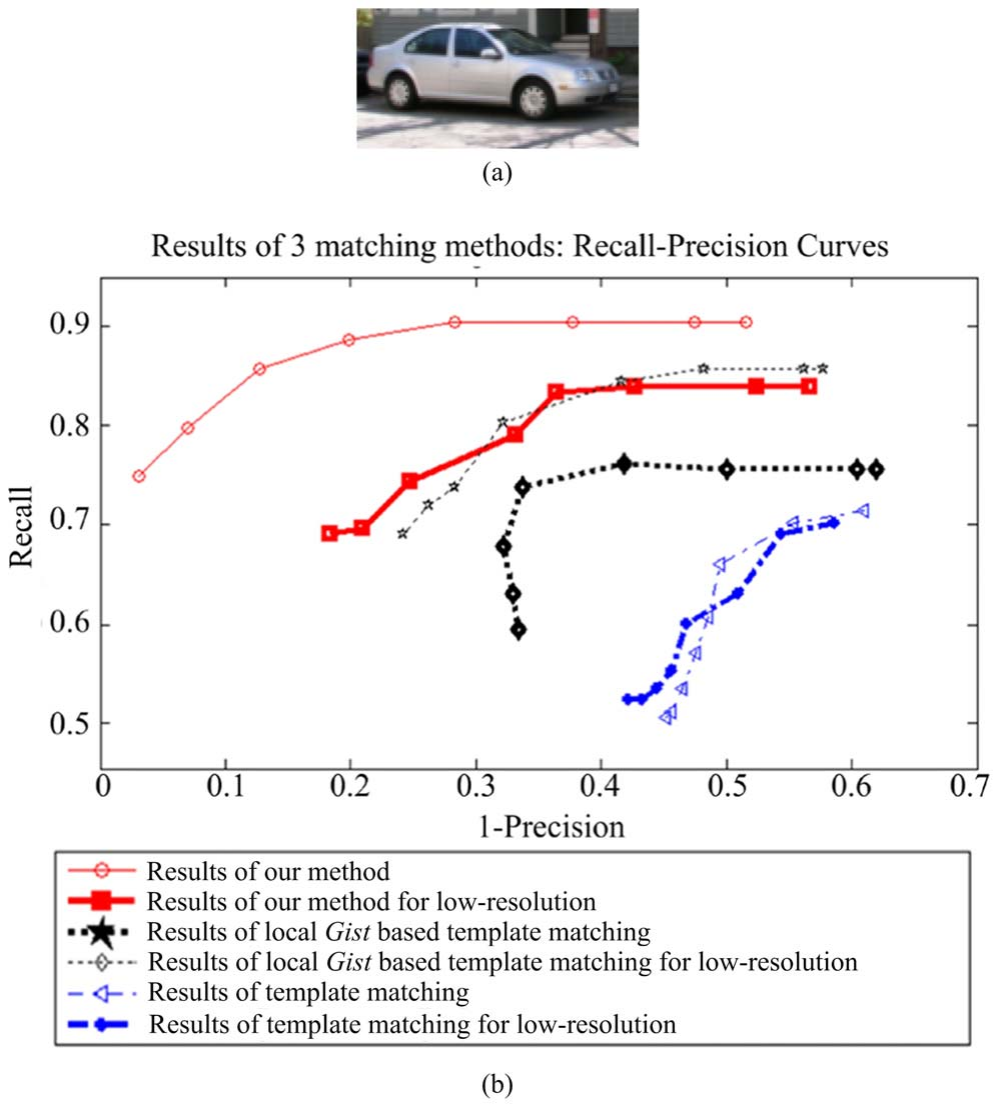
Recall-precision curves of template matching, local *Gist* based template matching, and our method on the LabelMe database. The results for low-resolution images are also given. The results show that our approach outperforms others.

**Figure 12:**
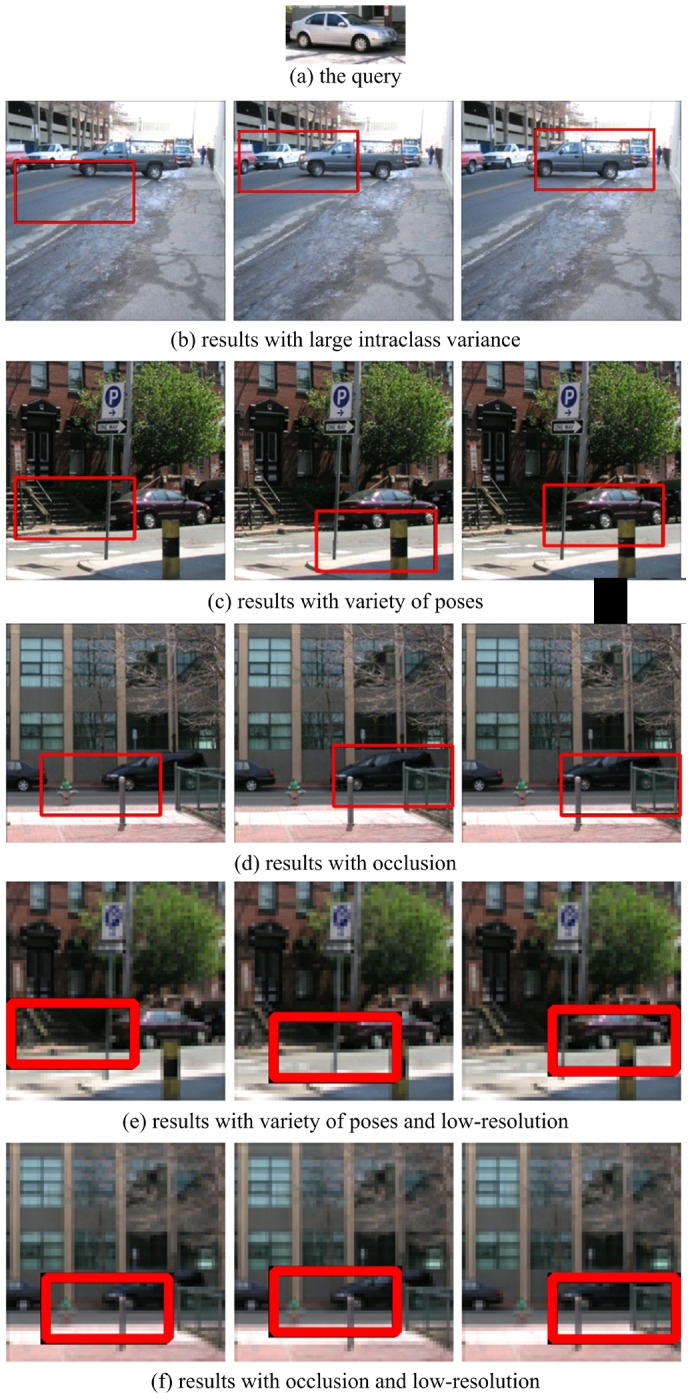
Car detection results with different challenges from the LabelMe dataset. For the subfigures (b)–(f ), the left images are the result of template matching; the middle images are the results of local *Gist* based template matching; and the right images are the results of our method.

We used 150 car images to evaluate the performance of the proposed method on the LabelMe database. These images were re-scaled so that the side view of the cars are roughly the same size (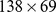). The template is from one of the scene images, as shown in Fig. 11(a). Fig. 11(b) presents the recall-precision curves of template matching, local gist based template matching, and our method, showing that our approach clearly outperforms others. The results for a 25% resolution conditions (the size of the object is 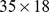) are given in Fig. 11(b). Some matching results are presented in Fig. 12. These results show the strong robustness of our method to different challenges.

### Experiments on UIUC

**Figure 13:**
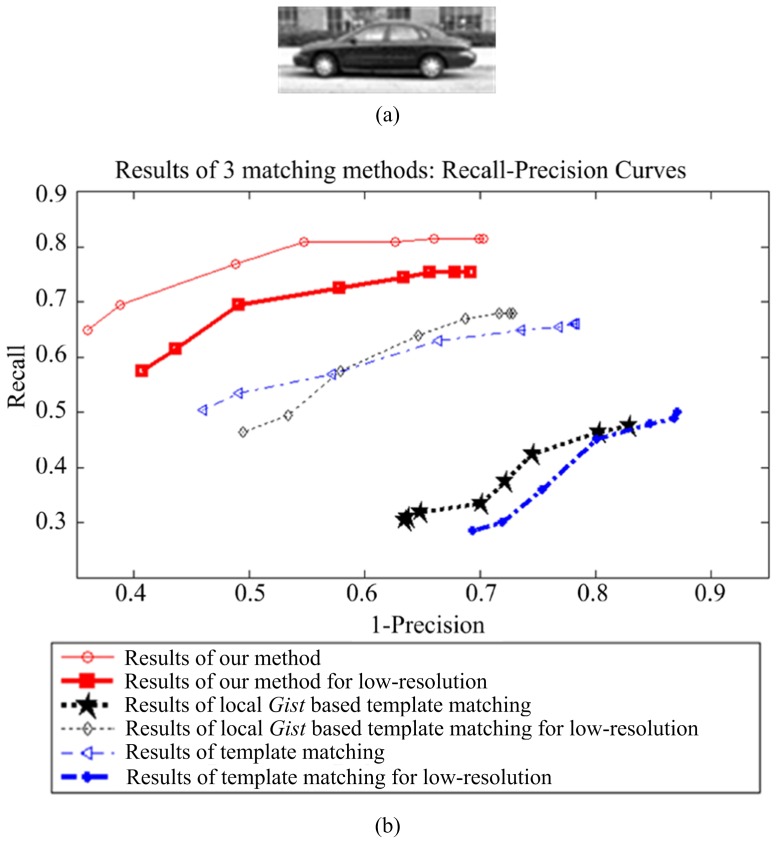
Recall-precision curves for template matching, local *Gist* based template matching and our method on the UIUC car database. The results show that our approach outperforms others.

**Figure 14:**
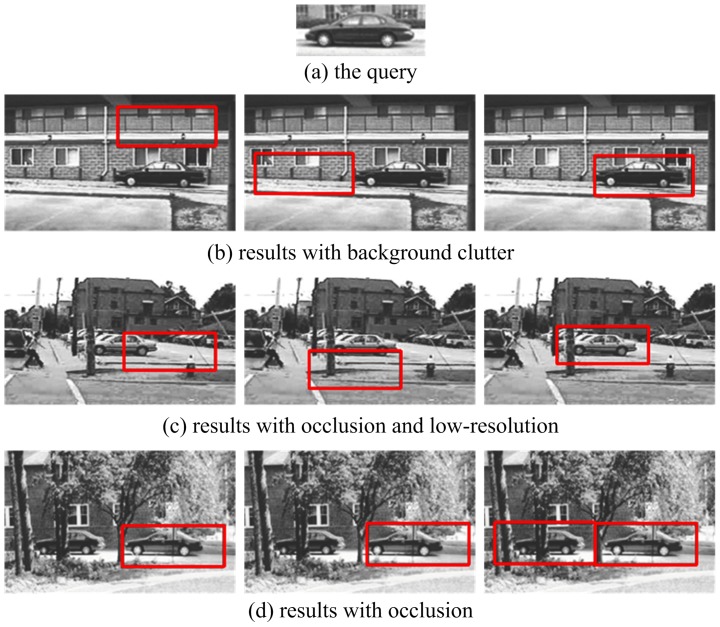
Car detection results with different challenges on the UIUC dataset. For the subfigures (b),(c), and (d), the left images are the result of template matching; the middle images are the results of local *Gist* based template matching; and the right images are the results of the proposed method.

The UIUC car dataset consists of small (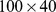 pixels) training images of 550 cars and 500 backgrounds. There are two sets of test images: a single-scale set in which the cars to be detected are roughly the same size as those in the training images, and a multi-scale set. Our approach can be expanded to multi-scale by searching at each scale, and we focus on the single-scale set in this work. The query template is from the training set on UIUC database, as shown in Fig. 13(a). Fig. 13(b) presents the results of template matching, local *Gist* based template matching and the proposed method as recall-precision curves, showing the good performance of our method. Results from a 25% resolution condition (the size of the object is 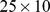) are also given in Fig. 13(b). Some matching results are presented in Fig. 14, which show that our approach is more robust.

We also report the Recall-Precision equal (RPC-equal, i.e., when recall  =  precision) error rates of the three methods together with that of local HoG [46] based template matching and Agarwal et al. [28] in Table 3. It can be seen that, our method outperforms local HoG based template matching method. The comparison results show that our method can compare with the template matching method with some powerful local representations, such as HoG. And we can see that the RPC-equal error rates of our method and the method in Ref. [28] are about the same. Note that, the latter method is learning based. The results demonstrate that for object detection tasks, the performance of the proposed method using only one object sample is very promising.

**Table 3:** The RPC-Equal Error Rates of our approach on the UIUC dataset and the comparison with template matching method, local *Gist* based template matching method, local HoG based template matching method, and the approach proposed by Agarwal et al. [28].

Methods	RPC-Equal error rate (%)
Template matching	60.4
Local *Gist* based template matching	59.1
The proposed method	**77.6**
Local HoG based template matching	62.7
Agarwal et al. [28]	76.5

The highest result is shown in bold.

### Experiments on two video sequences

**Figure 15:**
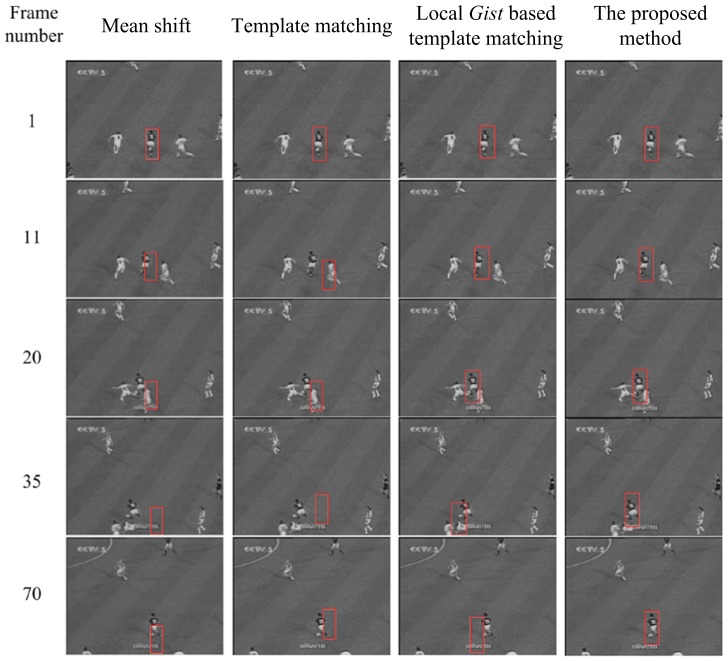
The tracking results for video 1 using the four methods: Mean shift, template matching, local *Gist* based template matching and our method. Five frames are presented, which are Frame 1, 11, 20, 35, 70 of the original video.

In this subsection, we report the tracking results on two video sequences, to demonstrate the robustness of the proposed method. For each frame, object tracking is very similar to the object detection problem, where the template is the detected result of the previous frame. The first video sequence is from a football match, where the player wearing No. 10 is the target in our tracking task. Tracking is over 70 frames. This task has several difficulties: the target is a non-rigid object; the player is partly occluded during the pass; and the background is moving. Fig. 15 presents the tracking results of template matching, local *Gist* based template matching, and our method. In addition, the results of the Mean shift method are also reported for comparison. Each row of the figure corresponds to a video frame, where the frame number is denoted at the far left. The tracked locations of the four methods on the frame are shown in different columns.

**Figure 16:**
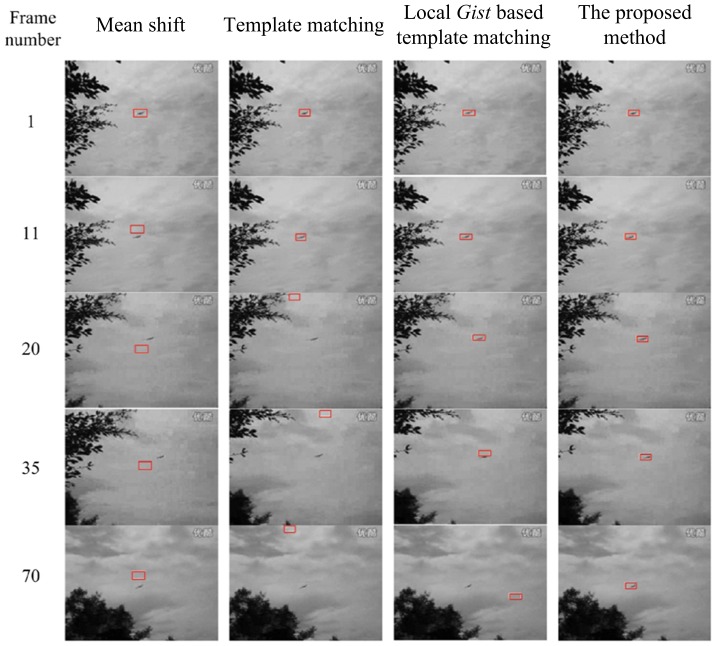
The tracking results for video 2 using the four methods: Mean shift, template matching, local *Gist* based template matching and our method. Five frames are presented, which are Frame 1, 5, 20, 30, 70 of the original video.

In the second video sequence, a plane flies in the sky, which is the object of interest. The length of the original sequence was 144 frames. Tracking was initiated in frame 1 and carried out for 70 frames (the plane is in shot for this range of frames). The plane in the video sequence is hard to track with a template matching method, because the plane is very small and hard to identify, and the background is also moving. Fig.16 presents the tracking results of the Mean shift method and the three methods mentioned above. The layout of Fig.16 is identical to that of Fig.15, where each row corresponds to a frame and each column corresponds to a tracking method.

We also report the number of continually tracked frames to evaluate the performance of these methods, as shown in Table 4. These results demonstrate the effectiveness and robustness of our approach for object tracking.

**Table 4:** The number of continually tracked frames (CTF).

Methods	CTF on the first video	CTF on the second video
Mean shift	4	2
Template matching	2	18
Local *Gist* based template matching	28	27
The proposed method	70	70

### Evaluation of generalization ability

**Figure 17:**
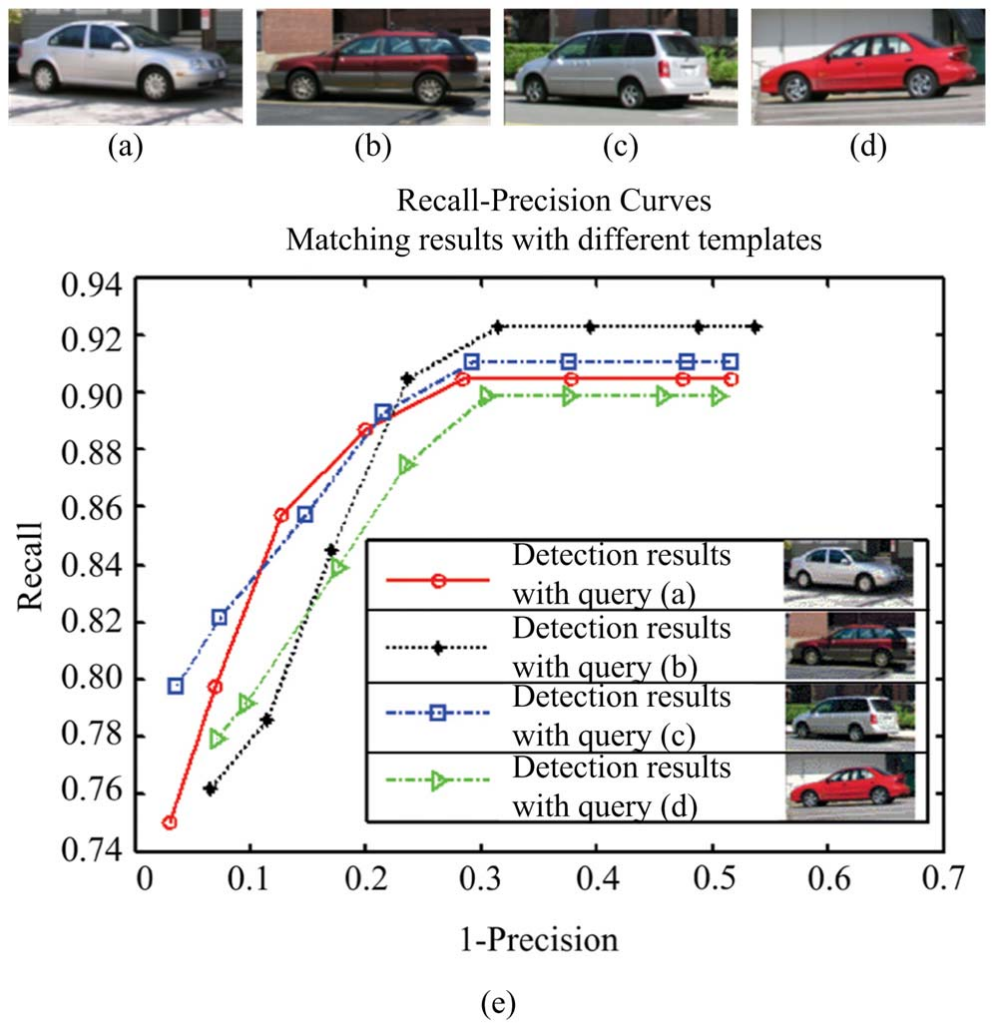
Comparison of performance of our method with 4 different query images on the LabelMe dataset. (a) query 1; (b) query 2; (c) query 3; (d) query 4; (e) Recall-precision curves for our method with 4 different query templates.

**Figure 18:**
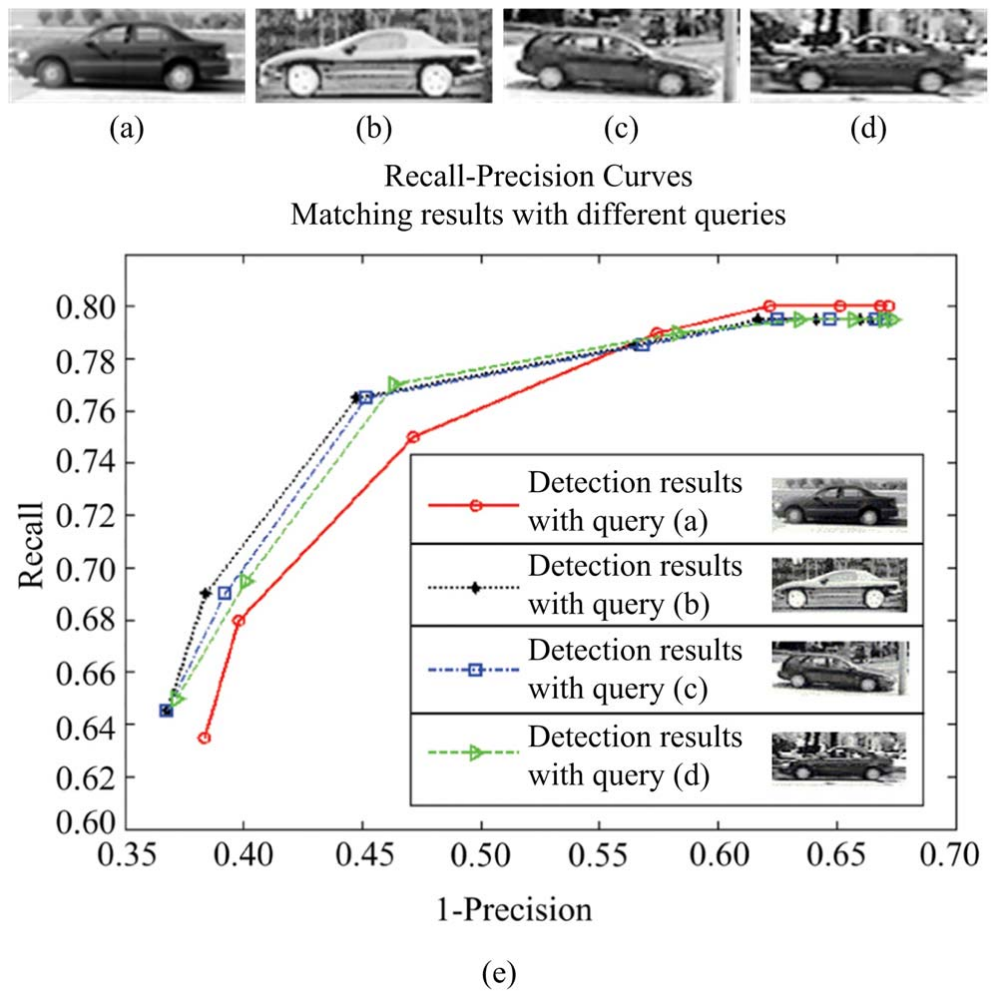
Comparison of performance of our method with 4 different query images on the UIUC car dataset. (a) query 1; (b) query 2; (c) query 3; (d) query 4; (e) Recall-precision curves for our method with 4 different query templates.

In addition, to analyze the effect of different query images on the performance of our method, we give the recall-precision curves of our method with 4 different query images in Fig. 17 and Fig. 18, on LabelMe and UIUC databases respectively. It can be seen that the detection performance is very close with different query templates. For in-depth analysis, the statistical results (RPC-equal error rates) of our approach with 100 different query templates are reported in Table 5 for both databases. The well-generalized results demonstrate that our approach does not depend on the particularity of queries.

**Table 5:** Statistical results (RPC-equal error rates) of our method with 100 different query images on the LabelMe dataset and the UIUC car dataset.

Statistical values	LabelMe	UIUC
Mean	91.2	79.5
Standard deviation	0.68	0.34

## Conclusions

In this paper, we have proposed an object detection method using single instances, which measures the similarity of a query and a sub-image in the scene by replacing the sub-image with the query and measuring the change in global scene context based on the *Gist* representation. And we have demonstrated the effectiveness of the proposed method with a variety of experiments. Using scene context, the proposed approach is more robust for object localization, including both detection in static images and tracking in dynamic scenes. Experiments show that our approach outperforms other conventional methods, especially when the queried object may show large intra-class variations, occlusions, various poses, low resolution, or be on a cluttered background.

We believe that the good performance of the proposed method is fostered by the scene context based representation, which describes the coarse layout information of the scene. It suggests that global representation is more robust and an object localization/detection method can be fragile without top-down contextual guidance.

In our current implementation, the method has some limitations: it is sensitive to scale and rotation since we did not take these factors into consideration, which should be relatively easy to incorporate. And our further research will be devoted to removing this limitation.
